# Brain function assessment of acupuncture for chronic insomnia disorder with mild cognitive dysfunction based on fNIRS: protocol for a randomized controlled trial

**DOI:** 10.3389/fneur.2024.1403785

**Published:** 2024-11-20

**Authors:** Tianyu Wang, Zhi Li, Tingting Ma, Fengya Zhu, Bin Yang, Sieun Kim, Runqing Miao, Jie Wu

**Affiliations:** ^1^Acupuncture and Tuina School, Chengdu University of Traditional Chinese Medicine, Chengdu, China; ^2^Department of Preventive Treatment, Hospital of Chengdu University of Traditional Chinese Medicine, Chengdu, China

**Keywords:** chronic insomnia disorder, mild cognitive dysfunction, acupuncture, randomized controlled trial, functional near-infrared spectroscopy, protocol

## Abstract

**Background:**

Chronic Insomnia Disorder (CID) is highly prevalent among older adults and impairs cognitive function. Insomnia accelerates the progression of mild cognitive impairment (MCI) and increases the risk of developing dementia. Acupuncture has been demonstrated in improving sleep quality and cognitive function. This study aims to explore the functional brain characteristics of CID with MCI patients and to assess the effects of acupuncture therapy using functional near-infrared spectroscopy (fNIRS).

**Methods and design:**

This study is a single-center randomized controlled trial. Participants will be randomly assigned to the manual acupuncture group or the placebo acupuncture group for an 8-week intervention period. fNIRS data will be collected during resting test and working memory test at baseline and at end of the intervention. The primary outcome is the change of the Montreal Cognitive Assessment (MoCA) score, secondary outcomes include the change of Mini-Mental State Examination (MMSE), Insomnia Severity Index (ISI), Patient Health Questionnaire-9 (PHQ-9), Generalized Anxiety Disorder Scale (GAD-7), and Apathy Evaluation Scale-Informant (AES-I).

**Discussion:**

The results of the study will provide insights into the effects of acupuncture on sleep quality and cognitive performance in CID with MCI patients. By utilizing fNIRS technology, we will elucidate the neural functional characteristic underlying the therapeutic benefits of acupuncture.

**Clinical trial registration:**

https://ClinicalTrials.gov, identifier ChiCTR2300076182.

## 1 Introduction

The burden of dementia is expected to increase rapidly due to the aging population. It is estimated that more than 150 million people will suffer from dementia by 2050. Efforts have been focused on the pre-dementia stage, known as mild cognitive impairment (MCI). Identifying potential risk factors is a crucial strategy to reduce the incidence of dementia. Chronic insomnia disorder (CID) affecting approximately 10–20% of the population, with older adults being particularly susceptible (1). Researchers found that chronic insomnia is a critical risk factor for cognitive decline. Older adults with insomnia face a greater risk of MCI compared to those with normal sleep patterns. MCI affects memory, language comprehension, cognitive processing, and decision-making abilities. It represents a stage preceding dementia. Abnormal sleep patterns disrupt biochemical and physiological functions associated with the nervous system (2). Evidence supports that chronic insomnia is an independent risk factor for cognitive decline (3), leading to neuroinflammation, oxidative stress, neuronal apoptosis, and amyloid-*β* (Aβ) buildup, which contribute to memory impairment and cognitive decline (4, 5). Recent studies have emphasized the link between sleep quality and brain aging. Chronic insomnia leads to cortical gray matter decline and subcortical structural atrophy, potentially contributing to the onset of MCI (6).

Short-term conversion rates from MCI to dementia range from 20 to 40%, while long-term rates over 5–10 years reach 60–100% (7–9). It is essential to prevent the onset and implement early interventions to delay its progression. Medications for dementia and other neurodegenerative diseases, including cholinesterase inhibitors, N-methyl-D-aspartate (NMDA) receptor antagonists, neurotrophic promoters, antioxidants, and anti-inflammatory drugs. However, the high costs and severe side effects resulting in low patient adherence (10). A trial showed the efficacy of a specific medication, Lecanemab, noted adverse events such as cerebral edema, infusion reactions, atrial fibrillation, syncope, and angina pectoris (11).

Acupuncture has shown effects in enhancing sleep quality and cognitive function, reducing fatigue, regulating emotions, and improving quality of life (12–14). It protects neural function by alleviating inflammation, modulating metabolism, controlling memory-related factors, and activating the brain cortex to reduce neuronal apoptosis (15–17). Acupuncture is one of the most often used adjuvant therapies for MCI patients (12). It has been widely used to improve cognitive function, with many studies confirming its efficacy (14–16). according to a previous NMA study, acupuncture may be the efficacy and safe complementary and alternative therapy for improving cognitive function in people with MCI (13).

In early stage, patients may not exhibit obvious brain structure changes, but neural network function has already altered. Thus, it is essential to explore the mechanisms from the perspective of brain function. Advances in imaging techniques and acupuncture research have led to increased exploration of the brain’s functional mechanisms affected by acupuncture therapy for neurodegenerative diseases (18). Reviews have noted widespread abnormalities in brain network connectivity and damage in brain regions during cognitive decline (19). Observations via neuroimaging indicate that acupuncture activates cortical functional brain areas, enhances blood flow, regulates brain networks, and improves visual–spatial function (20).

Functional near-infrared spectroscopy (fNIRS) detects changes in brain function by measuring the absorption of near-infrared light by oxygenated (HbO) and deoxygenated hemoglobin (HbR). It provides high resolution without invasive procedures or significant motion interference (21, 22). This technology facilitates monitoring of brain region activity and connectivity (23, 24). Previous studies (25, 26) have found changes in brain functional imaging in insomnia and cognitive decline individuals, and acupuncture could regulate these changes (27). Prior study has confirmed that CID patients exhibit working memory deficits related to cognitive levels with altered connectivity patterns change between the frontal and temporal regions. It possibly serving as the neural basis of cognitive impairment (28). Additionally, acupuncture can modulate network patterns in the frontal and temporal lobes of insomnia patients (29). Evidence supports that acupuncture is benefit to sleep quality and cognitive enhancement, but brain imaging characteristics and solid proof from clinical trials are lacking. Based on the background, this study aims to investigate the brain functional characteristics of CID with MCI patients using fNIRS technology, and assess the changes following acupuncture intervention.

## 2 Objectives

This study aims to assess the activation patterns and functional connectivity in the frontal and temporal brain regions of CID with MCI patients and identify the effects of acupuncture on brain function related to sleep and cognitive performance.

## 3 Methods

### 3.1 Study design

This is a randomized, single-blind, placebo controlled clinical trial. This study will be conducted at the Affiliated Hospital of Chengdu University of Traditional Chinese Medicine. Reporting of the protocol will adhere to the Standards for Reporting Interventions in Clinical Trials of Acupuncture (STRICTA) guidelines (30) (Table 1) and the Consolidated Standards of Reporting Trials (CONSORT) 2010 Checklist (31) (Table 2).

**Table 1:** STRICTA 2010 checklist of information to include when reporting interventions in a clinical trial of acupuncture (expansion of item 5 from CONSORT 2010 checklist).

Item	Item number	Detail
1. Acupuncture rationale	(1a) Style of acupuncture (e.g., Traditional Chinese Medicine, Japanese, Korean, Western medical, Five Element, ear acupuncture, etc.)	Traditional Chinese Medicine (TCM)
	(1b) Reasoning for treatment provided, based on historical context, literature sources, and/or consensus methods, with references where appropriate	The treatment is based on traditional Chinese medicine acupuncture theory, literature support, and expert consensus
2. Details of needling	(1c) Extent to which treatment was varied	Standardized acupuncture treatment
	(2a) Number of needle insertions per subject per session	Twelve
	(2b) Names of points used	GV20 (Baihui), EX-HN1 (Sishencong), GV24 (Shenting), HT7 (Shenmen), GB39 (Xuanzhong), and KI4 (Dazhong)
	(2c) Depth of insertion, based on a specified unit of measurement, or on a particular tissue level	From 10 to 40 mm
	(2d) Response sought (e.g., de qi or muscle twitch response)	Deqi (soreness, tingling, heaviness, distention, etc.)
	(2e) Needle stimulation (e.g., manual, electrical)	Manual acupuncture
	(2f) Needle retention time	30 min
	(2 g) Needle type (diameter, length, and manufacturer or material)	Sterile, disposable acupuncture needles (diameter, 0.25 mm; length,25/40 mm; Hwato, China)
3. Treatment regimen	(3a) Number of treatment sessions	24 sessions
	(3b) Frequency and duration of treatment sessions	3 sessions a week (every other day), for a total of eight weeks
4. Other components of treatment	(4a) Details of other interventions administered to the acupuncture group (e.g., moxibustion, cupping, herbs, exercises, lifestyle advice)	Track the regular usage of sleep medications and provide guidance for daily living
	(4b) Setting and context of treatment, including instructions to practitioners, and information and explanations to patients	The trial will be conducted at the Department of Preventive Treatment at Chengdu University of TCM. Researchers will diagnose and give informed consent to the participants, the acupuncturist will be responsible for providing treatment and lifestyle advice, and designated researchers will be responsible for randomization and data collection. All participants of the acupuncture group will be arranged to the acupuncture room for treatment
5. Practitioner background	Description of participating acupuncturists (qualification or professional affiliation, years in acupuncture practice, other relevant experience)	Trained, licensed acupuncturists with at least 2 years in acupuncture clinical practice
6. Control or comparator interventions	(6a) Rationale for the control or comparator in the context of the research question, with sources that justify this choice	The aim of setting the placebo acupuncture as a control group is to explore the specific effects of manual acupuncture on participants
	Precise description of the control or comparator. If sham acupuncture or any other type of acupuncture-like control is used, provide details as for items 1 to 3 above	In the control group, operators stimulate the same acupoints as the manual acupuncture group for 8 weeks, 3 sessions a week (every other day), with the use of PSD + retractable blunt needles. Except for the operators, every one of the participants in the study is blind to the intervention.

**Table 2:** CONSORT 2010 checklist with the Non-pharmacological Trials Extension to CONSORT (with STRICTA 2010 extending CONSORT Item 5 for acupuncture trials)

Section/Topic	Item	CONSORT 2010 Statement: Checklist item	Page
TITLE AND ABSTRACT			
	1.a	Identification as a randomized trial in the title	1
	1.b	Structured summary of trial design, methods, results, and conclusions; for specific guidance see CONSORT for Abstracts	1
INTRODUCTION			
Background and objectives	2.a	Scientific background and explanation of rationale	2
	2.b	Specific objectives or hypotheses	2
METHODS			
**Trial design**	3.a	Description of trial design (e.g., parallel, factorial) including allocation ratio	2
	3.b	Important changes to methods after trial commencement (e.g. eligibility criteria), with reasons	5
Participants	4.a	Eligibility criteria for participants	5
	4.b	Settings and locations where the data were collected	5
**Interventions**	5	The interventions for each group with sufficient details to allow replication, including how and when they were actually administered	6-7
Outcomes	6.a	Completely defined pre-specified primary and secondary outcome measures, including how and when they were assessed	7-9
	6.b	Any changes to trial outcomes after the trial commenced with reasons	10-11
Sample size	7.a	How sample size was determined	3
	7.b	When applicable, explanation of any interim analyses and stopping guidelines	5
Randomization			
Sequence generation	8.a	Method used to generate the random allocation sequence	5
	8.b	Type of randomization; details of any restriction (e.g., blocking and block size)	5
Allocation concealment	9	Mechanism used to implement the random allocation sequence (e.g., sequentially numbered containers), describing any steps taken to conceal the sequence until interventions were assigned	5
Implementation	10	Who generated the random allocation sequence, who enrolled participants, and who assigned participants to interventions	12
**Blinding**	11.a	If done, who was blinded after assignment to interventions (e.g. participants, care providers, those assessing outcomes) and how	5
	11.b	If relevant, description of the similarity of interventions	6-7
**Statistical methods**	12.a	Statistical methods used to compare groups for primary and secondary outcomes	10-11
	12.b	Methods for additional analyses, such as subgroup analyses and adjusted analyses	10-11
RESULTS			
Participant flow (A diagram is strongly recommended)	13.a	For each group, the numbers of participants who were randomly assigned, received intended treatment, and were analyzed for the primary outcome	2-3
	13.b	For each group, losses and exclusions after randomization, together with reasons	2-3
Implementation of intervention			
Recruitment	14.a	Dates defining the periods of recruitment and follow-up	9
	14.b	Why the trial ended or was stopped	6
**Baseline data**	15	A table showing baseline demographic and clinical characteristics for each group	not applicable
Numbers analyzed	16	For each group, number of participants (denominator) included in each analysis and whether the analysis was by original assigned groups	10
Outcomes and estimation	17.a	For each primary and secondary outcome, results for each group, and the estimated effect size and its precision (e.g., 95% confidence interval)	10
	17.b	For binary outcomes, presentation of both absolute and relative effect sizes is recommended	10
Ancillary analyses	18	Results of any other analyses performed, including subgroup analyses and adjusted analyses, distinguishing pre-specified from exploratory	10
Harms	19	All important harms or unintended effects in each group; for specific guidance see CONSORT for Harms [60]	9-10
DISCUSSION			
Limitations	20	Trial limitations, addressing sources of potential bias, imprecision, and, if relevant, multiplicity of analyses	12
Generalizability	21	Generalizability (external validity, applicability) of the trial findings	11-12
Interpretation	22	Interpretation consistent with results, balancing benefits and harms, and considering other relevant evidence	11-12
Other information			
Registration	23	Registration number and name of trial registry	1
Protocol	24	Where the full trial protocol can be accessed, if available	1
Funding	25	Sources of funding and other support (e.g., supply of drugs); role of funders	13

### 3.2 Ethics approval

This trial protocol has been approved by the Ethics Committee of the Affiliated Hospital of Chengdu University of Traditional Chinese Medicine (2023KL-063), and registered in https://ClinicalTrial.gov (Identifier: ChiCTR2300076182). The study is performed according to the Helsinki Declaration. Permission will be obtained from hospital before conducting. The Ethics Committee will supervise and review the design and implementation of the trial methods.

### 3.3 Sample size calculation

According to a prior clinical study (32), sample size estimation is based on the Montreal Cognitive Assessment (MoCA) score. The differences in MoCA scores before and after treatment was (3.22 ± 1.88) for the acupuncture group and (1.97 ± 1.77) for the placebo acupuncture group. In the formula, with *α* = 0.05, *β* = 0.10, and k = 1, a sample size of 37 cases per group was calculated. Considering a dropout rate of 20%, we determined a sample size of 45 cases per group. Consequently, the total number of cases in both groups will be 90.

Using brain imaging data as an indicator of acupuncture-related studies, the sample typically ranged from 10 to 25 individuals per group (33–35). Thus, we will enroll at least 20 participants per group in fNIRS scanning. Considering a dropout rate of 20%, we determined a sample size of 24 cases per group.

$$n=\frac{{\left(u_{\alpha }+\mu_{\beta }\right)}^{2}\left(1+1∕k\right)\sigma^{2}}{\delta^{2}}$$

### 3.4 Participants

This trial is aimed at aging individuals diagnosed as CID with MCI. Participants will be recruited from the clinical center. Eligible participants will be randomly divided into either an 8-week manual acupuncture or placebo acupuncture group.

### 3.5 Recruitment and procedure

**Figure 1:**
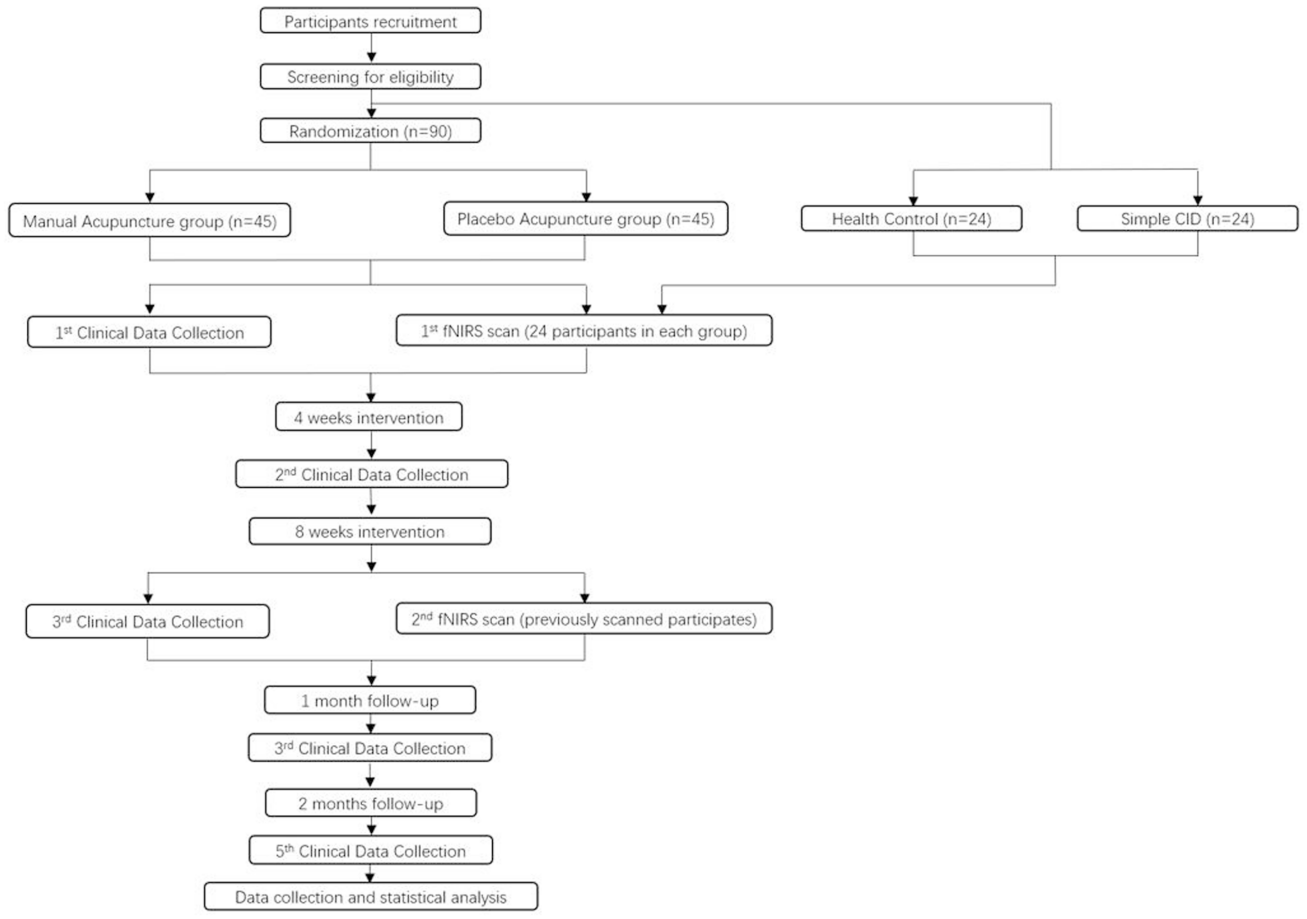
The flowchart of trial procedures.

The recruitment will be conducted through advertisements and online platforms. Participants will be recruited from the Preventive Healthcare Center at the Affiliated Hospital of Chengdu University of Traditional Chinese Medicine. Recruitment efforts will involve posting posters in Preventive Healthcare Center and promotional content on the WeChat platform, outlining information such as the target patient population, eligibility criteria, interventions, and contact details. Participants will be provided study brochure and requested to fill up screening form. Patients will be required to sign a informed consent form if they meet the eligibility and agree to participate. Outcomes will be evaluated at baseline, 4 weeks (mid-intervention), 8 weeks (post-intervention), and follow-up period. In each group, 24 individuals will be selected for fNIRS scanning before and after intervention. Additionally, 24 healthy participants and 24 insomnia patients without cognitive impairment will be included as controls for assessing the brain functional characteristics of CID with MCI patients before intervention (Figure 1).

### 3.6 Diagnostic criteria

The criteria for diagnosing CID are as follows: (1) meeting the diagnostic standards in the International Classification of Sleep Disorders (ICSD); (2) experiencing difficulty falling asleep, maintaining sleep, or waking up early on at least 3 nights per week for a duration of 3 months; and (3) an inability to attribute these sleep difficulties to other sleep disorders.

The criteria for diagnosing MCI are as follows: (1) the patient or a reliable informant reports cognitive impairment or a clinician with experience detects it; (2) impairment is observed in cognitive domains (memory, language, visuospatial, and executive functioning); (3) the patient can perform daily activities without assistance; and (4) dementia is not present.

### 3.7 Eligibility criteria

Participants will be recruited if they meet the following criteria: (1) conform to the ICSD-3 diagnostic criteria for CID and Pittsburgh Sleep Quality Index (PSQI) score > 7; (2) cognitive impairment reported by the caregiver and confirmed by an experienced clinician; (3) impairment is observed in any aspect of memory, language, visuospatial, or executive functioning; (4) aged between 50 and 75 years, any gender, right-handed; (5) ongoing daily activities without assistance; (6) without dementia; (7) without recent acupuncture interventions (at least 1 month); (8) capable of effective communication and cooperation without severe visual or hearing impairment; and (9) willing to enroll and provide informed consent.

### 3.8 Exclusion criteria

The exclusion criteria are as follows: (1) severe cardiovascular, cerebrovascular, hepatic, renal, infectious, malignant, or hematological diseases, as well as any severe conditions that could impact trial outcomes or increase participation risk; (2) cognitive dysfunction secondary to other conditions, such as coronary heart disease, chronic obstructive pulmonary disease, head trauma, stroke, etc.; (3) pregnancy or lactation; (4) allergies to metal or adhesive; (5) severe skin lesions at the needling site; (6) participation in other research projects; (7) inability to schedule suitable participation times or lack of sufficient willingness or confidence to complete the trial; and (8) previous receipt of acupuncture.

### 3.9 Termination criteria

The termination criteria are as follows: (1) Severe safety issues arise; (2) The treatment is ineffective, lacking clinical value; (3) Significant errors in the clinical trial protocol or major deviations occur during implementation; (4) The project applicant requests termination (e.g., due to funding reasons, management reasons, etc.); and (5) Administrative authorities revoke the trial.

### 3.10 Randomization and blinding

Participants will be randomly assigned to two groups in a 1:1 ratio using computer-generated random numbers. During intervention phase, participants will be administered separately in quiet treatment rooms to prevent interactions from different groups. Throughout the study, researchers, acupuncturist, efficacy assessors, and data statisticians will remain segregated. Only the acupuncturist aware of the group assignment result. Participants, evaluators, and analysts will be blinded to group assignments.

### 3.11 Data monitoring committee

The Data Monitoring Committee (DMC) for this study comprised independent experts with no direct involvement in the research project. The committee included Dr. Rongli Yuan, who provided expertise in data analysis and statistical oversight, and Dr. Mengjing Wang, who contributed to evaluating safety and efficacy from an acupuncture perspective.

The DMC was responsible for monitoring safety and efficacy data, reviewing interim results, and making recommendations regarding the continuation, modification, or termination of the trial. The committee operated independently from both the research team and the ethics committee to ensure objective oversight.

## 4 Intervention

Following the STRICTA guidelines (30), the treatment will focus on specific acupuncture points. The selection of these points was based on traditional Chinese medicine theory, supplemented by evidence from prior research findings, including systematic reviews and meta-analyses, data mining results (36–38), and the consensus of experienced experts within our research team. The selected acupoints include Baihui (GV20), Sishencong (EX-HN1), Shenting (GV24), Shenmen (HT7), Xuanzhong (GB39), and Dazhong (KI4).

**Figure 2:**
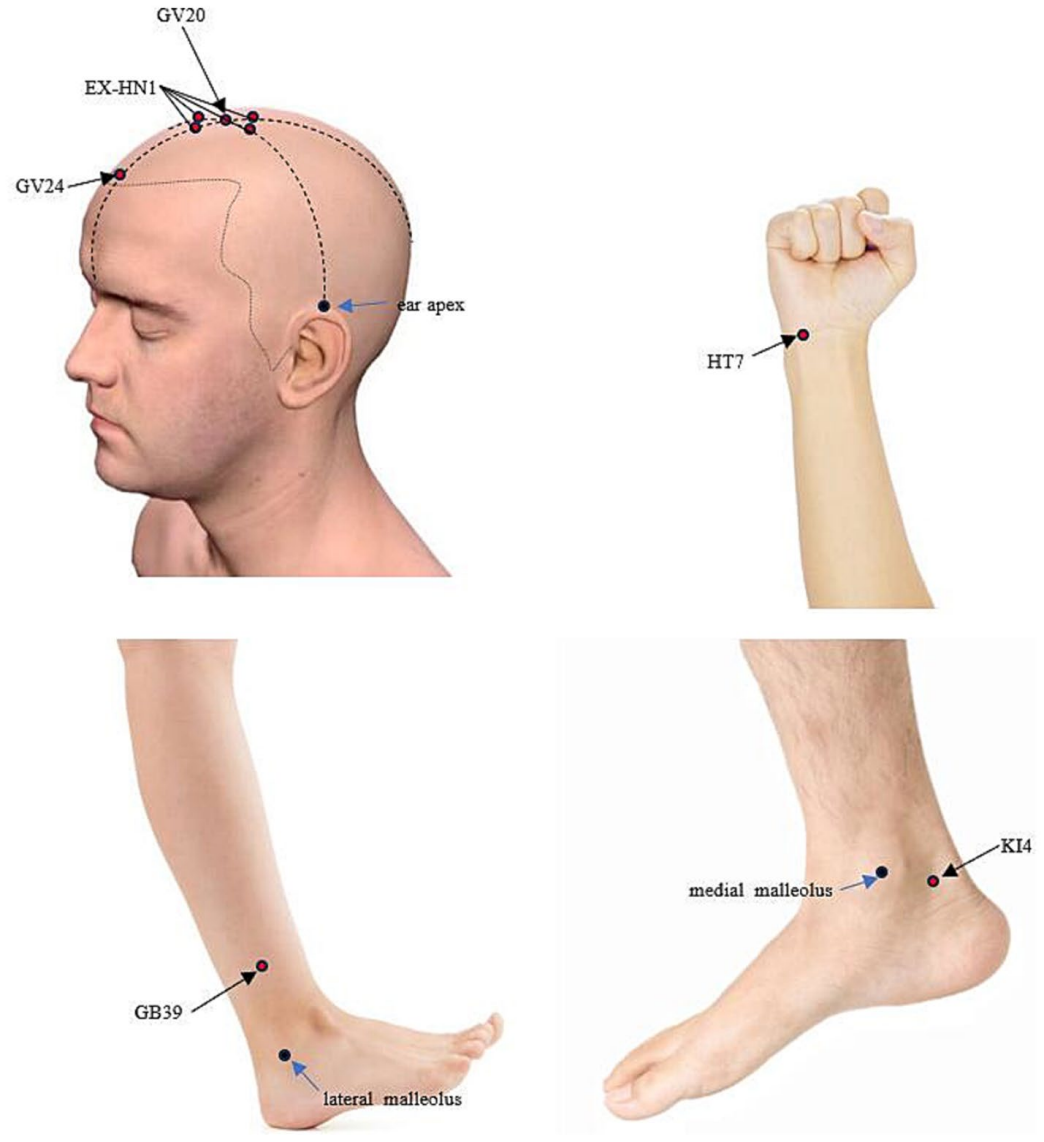
Localization of the applied acupoints. GV20: located at the highest point on the head, in the midline, directly on the apex of the head. EX-HN1: situated around GV20, with one point in each of the four directions: anterior, posterior, left, and right. These points are typically located about 1 cun (Chinese inch) away from GV20 in each direction. GV24: located on the Du Meridian, at a point 0.5 cun directly above the midpoint of the anterior hairline. HT7: located on the transverse crease of the wrist, on the ulnar aspect of the palm, in the depression between the tendons of the flexor carpi ulnaris and the flexor digitorum superficialis. GB39: located on the lateral side of the lower leg, about 3 cun above the external malleolus, near the anterior border of the fibula. KI4: located on the medial side of the foot, posteroinferior to the medial malleolus, at the upper edge of the calcaneus, in the depression at the anterior attachment of the achilles tendon.

**Figure 3:**
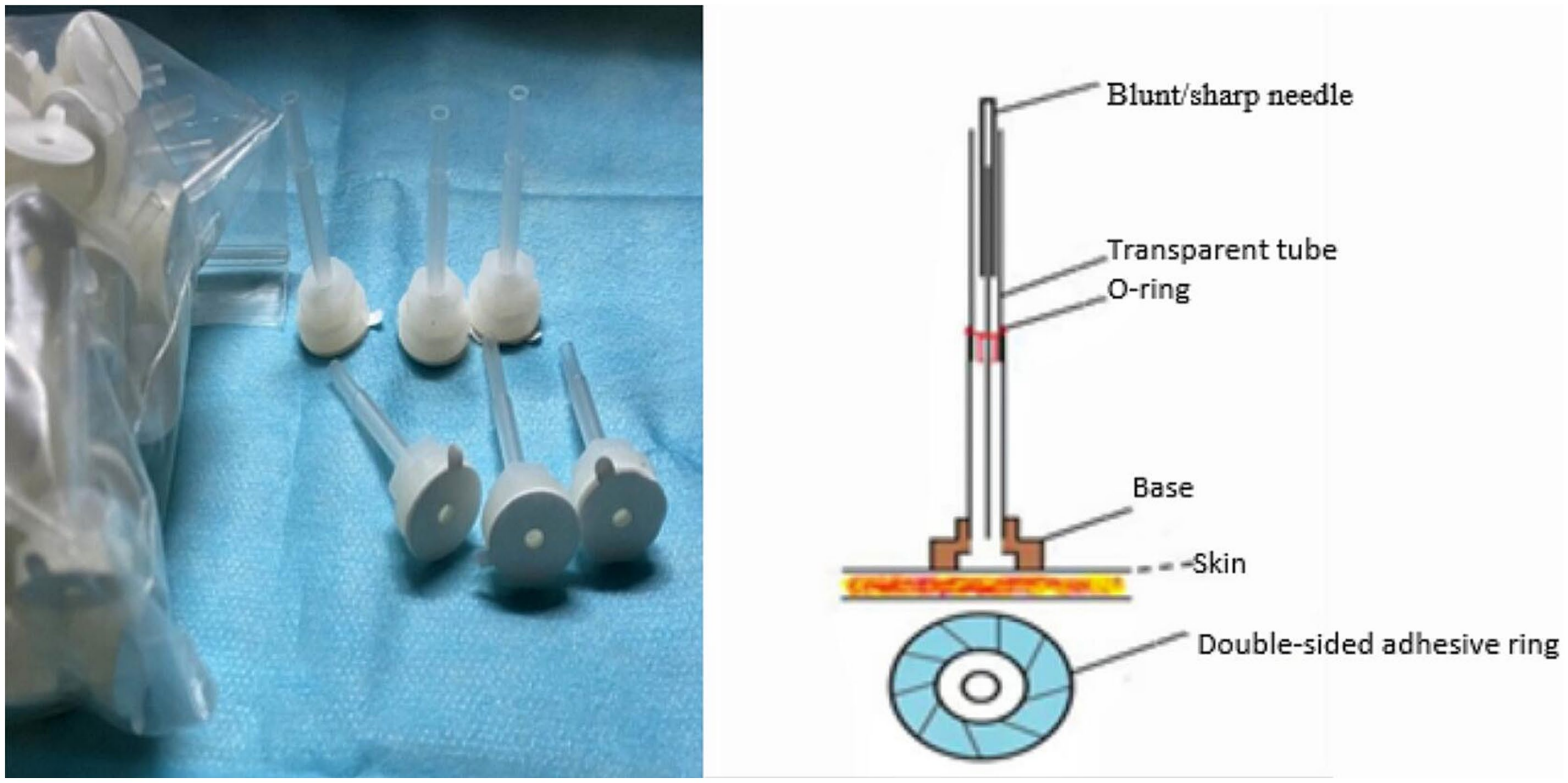
The device of manual or placebo acupuncture.

**Figure 4:**
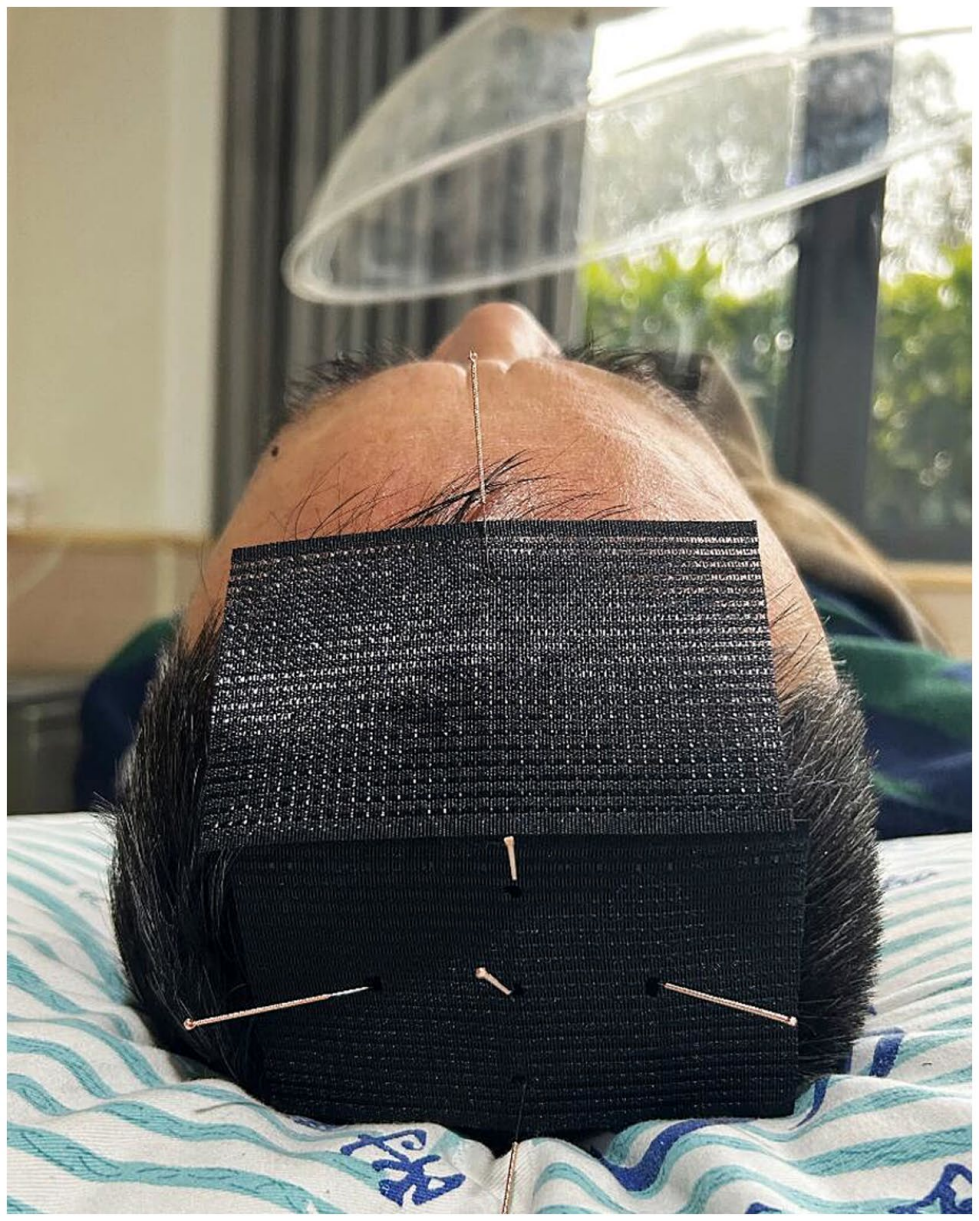
The specialized device of manual or placebo acupuncture for areas covered by hair.

The localization and manipulation of acupoints rooted in Traditional Chinese Medicine (TCM) standards (Figure 2). To ensure blinding, the Park Sham Device (PSD) will be utilized following skin disinfection. Adhesive tape will secure acupoints on extremities, while a hair gripper will be used for acupoints on the head (Figures 3, 4). Patients could receive basic supportive care or sleeping pill. Detailed such as duration, dose, frequency, and response will be recorded.

### 4.1 Manual acupuncture group

Participants in the manual acupuncture group will receive intervention with PSD + disposable sterile needles (Hwato, Suzhou, China; 0.25 × 25 mm). After disinfection, the PSD will be placed at the acupoints, disposable sterile needles will be quickly inserted into the skin using a pre-prepared tube. For acupoints on the head, including Baihui (GV20), Sishencong (EX-HN1), and Shenting (GV24), needles are inserted at a 15° angle to a depth of 0.5 cun (approximately 13 mm). For other acupoints, including Shenmen (HT7), Xuanzhong (GB39), and Dazhong (KI4), needles are inserted perpendicularly at a 90° angle to a depth of 1 cun (approximately 25 mm).

The practitioner will perform lifting, thrusting, or rotating techniques to elicit the “deqi” sensation, which indicates the therapeutic efficacy of acupuncture. “De qi” typically manifests as sensations of soreness, numbness, fullness, or radiance and helps practitioners confirm whether the needling technique is appropriate, ensuring treatment accuracy. Due to individual differences among participants, acupuncturists adjust their techniques based on each participant’s specific condition to achieve “deqi” sensation. The needles will then be retained for 30 min.

### 4.2 Placebo acupuncture group

Participants in the placebo acupuncture group will receive intervention with PSD + retractable blunt needles (AcuPrime; 0.25 mm × 25 mm). After placing the device, retractable blunt needles will be used to simulate needle insertion, creating the sensation of being stimulated by a needle without actually penetrating the skin. No lifting, thrusting, or rotating techniques will be performed to avoid generating the sensation of Deqi, and the needles will be retained for 30 min.

## 5 Outcome measures

Each participant will be evaluated by a medical professional blinded to the group assignment and intervention. Assessments will occur at five time points: baseline, 4 weeks, 8 weeks, 12 weeks, and 20 weeks. The schedule of this study is listed in Table 3.

**Table 3:** Study schedule.

Timepoint	Enrolment	Baseline	Treatment phase		Follow-up phase	
	1 week	0 week	4 weeks	8 weeks	1 month	3 months
Enrolment						
Eligibility screen	**×**					
Medical history	**×**					
Informed consent		**×**				
Allocation		**×**				
Interventions						
Manual acupuncture		**×**	**×**	**×**		
Placebo acupuncture		**×**	**×**	**×**		
Health education		**×**	**×**	**×**		
Assessments						
Demographics	**×**					
fNIRS scanning		**×**		**×**		
PSQI	**×**	**×**				
MoCA	**×**	**×**	**×**	**×**	**×**	**×**
ISI		**×**	**×**	**×**	**×**	**×**
MMSE		**×**	**×**	**×**	**×**	**×**
PHQ-7		**×**	**×**	**×**	**×**	**×**
GAD		**×**	**×**	**×**	**×**	**×**
AES		**×**	**×**	**×**	**×**	**×**
SF-12		**×**	**×**	**×**	**×**	**×**
Safety assessment		**×**	**×**	**×**		
Vital sign		**×**	**×**	**×**		
Use of sleep medications		**×**	**×**	**×**	**×**	**×**

### 5.1 Primary outcomes

#### 5.1.1 MoCA

MoCA is used to evaluate cognitive functioning, including attention, concentration, executive functioning, memory, expression, visual-structural skills, abstract thinking, computation, and orientation. The total score ranging from 18 to 25 indicates MCI. If the patient with no more than 12 years of education, then add 1 point to the total score.

### 5.2 Secondary outcomes

#### 5.2.1 MMSE

The Mini-mental state examination (MMSE) could evaluate memory function, and offers a rapid and accurate measure of intellectual status and cognitive deficits. The score adjusted based on education level, with cutoff criteria for normal cognitive functioning are as follows: more than 17 points for uneducated individuals, more than 20 points for low-educated individuals, and more than 24 points for highly educated individuals.

#### 5.2.2 ISI

Insomnia Severity Index Scale (ISI) is designed to evaluate insomnia symptoms experienced in the past 2 weeks. It consists of 7 items, comprising daytime function and nighttime symptoms. Each item is scored from 0 to 4. The criteria for insomnia are as follows: mild insomnia ranges from 8 to 14, moderate insomnia ranges from 15 to 21, and severe insomnia ranges from 22 to 28. This assessment tool is invaluable for evaluating the outcomes of interventions targeting insomnia.

#### 5.2.3 PHQ-9, GAD-7, and AES-I

Anxiety, depression, and apathy are recognized as risk factors for dementia. Considering of the characteristics of patients, we incline to use more concise, objective, and efficient scales in emotional assessment. The Patient Health Questionnaire-7 (PHQ-9) consists of 9 items, each item rated on 0–3 score. The Generalized Anxiety Disorder (GAD-7) scale comprises 7 items, and each item ratings from 0 to 3 to assess psychogenic anxiety symptoms. The Apathy Evaluation Scale-Informant (AES-I) consists of 18 items, each item rated on a scale of 0–3. It will be used to assess the apathy degree of patients. Higher scores indicate more severe symptoms of depression, anxiety, or apathy.

#### 5.2.4 SF-12

The Short Form 12 Health Survey (SF-12) scale includes 8 dimensions of 12 items. This scale is widely used in large-scale health measurement. The score is positively correlated with the level of health quality.

### 5.3 fNIRs data of brain function

**Figure 5:**
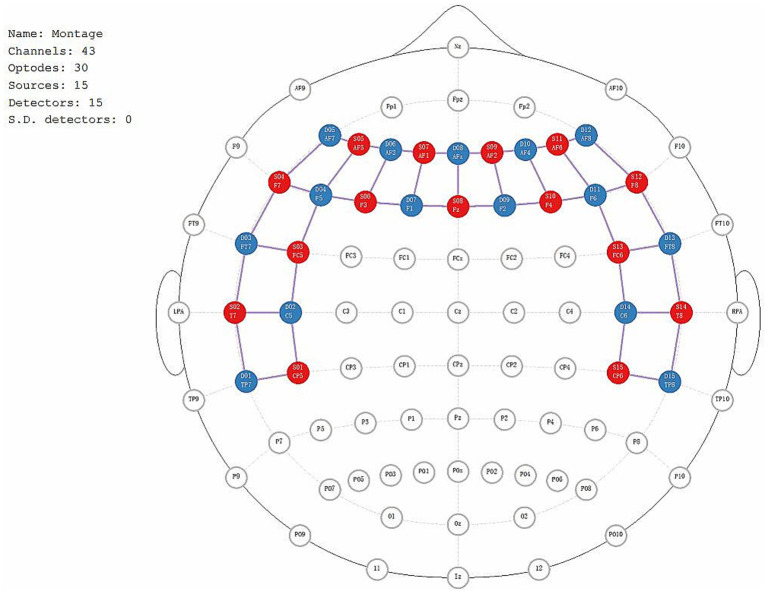
The positions of the emitters, detectors, and the 43 channels.

The fNIRS data will be recorded using the NIRx Imager system (NIRx Medical Technologies, Germany). Fifteen transmitters and fifteen detectors will be utilized to capture cerebral oxygenation signals. Initial scalp localization will be performed using established stereospecific and anatomical markers. The positions of the emitters, detectors, and the 43 channels covering the frontal and temporal lobes will be precisely identified (Figure 5). To capture the fNIRS data, the device will emit near-infrared light at wavelengths of 785 nm and 830 nm, with a continuous wave frequency of 3.096 Hz. Before the testing procedure, participants will be briefed on the study objectives and instructed to maintain optimal stillness of their heads throughout the testing process to minimize potential errors. The study procedure includes two parts: a resting test and an N-back working memory test.

**Figure 6:**
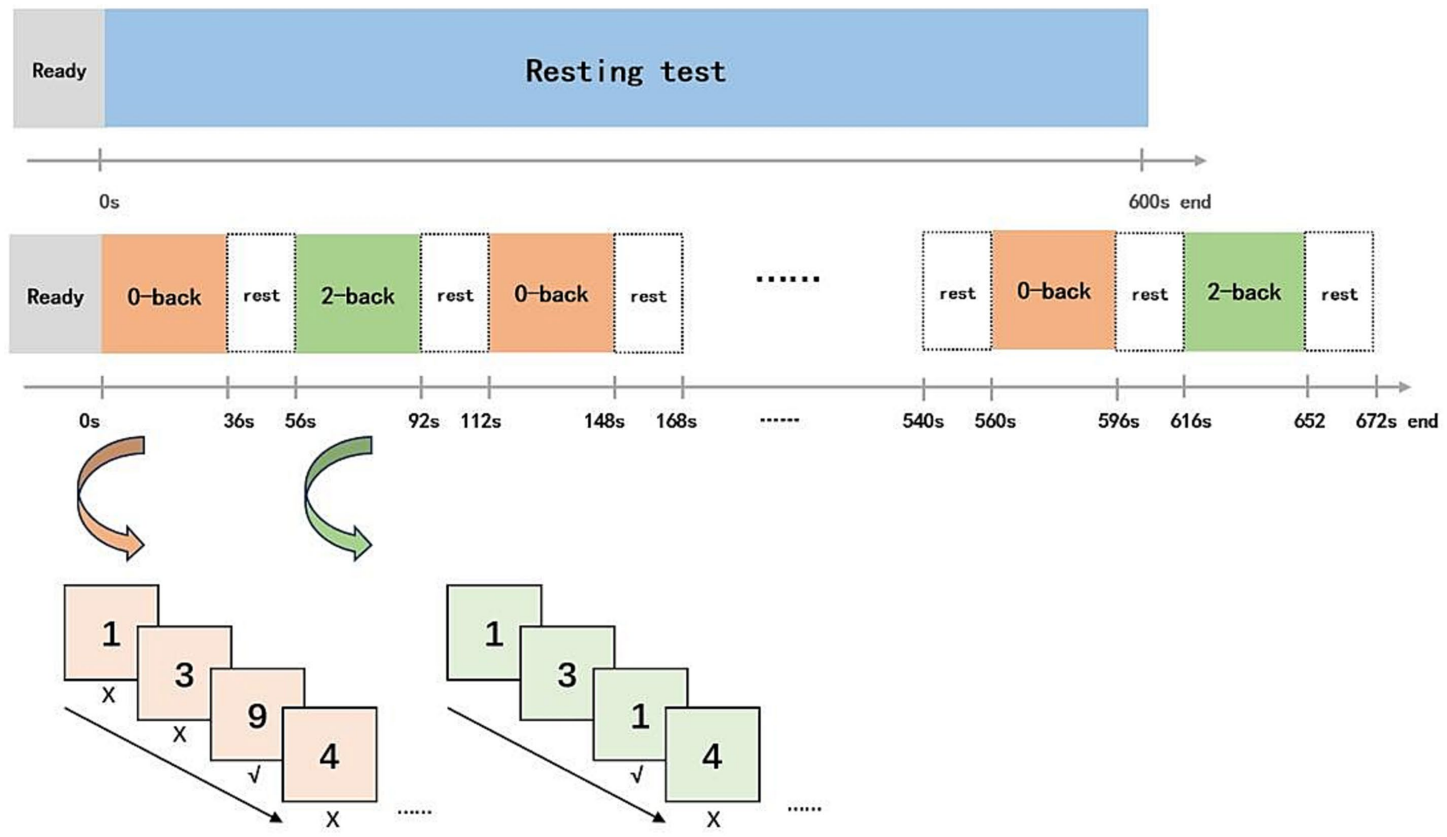
Resting test and working memory test processes.

During resting test, all anticipants will be required to remain seated with their eyes closed for 300 s. In the N-back working memory task, a block design alternating between 0-back and 2-back numerical tasks will be employed. In the 0-back task, 12 numbers will be presented randomly, and participants will be asked to identify whether the displayed number is “9” from a range of 0–9. In the 2-back task, participants will be required to determine whether the current number matches the one shown two positions earlier. The task sequence will follow a “0-2-0-2” pattern, with six blocks for each task type, and a 20 s interval marked by a “+” sign on the screen (Figure 6). The correct rate for the 0-back and 2-back tasks will be calculated to the total number of each task type.

## 6 Assessment of safety and “deqi” sensations

Vital signs including heart rate, blood pressure, respiration rate, and oxygen saturation will be monitored prior and following each intervention. Any adverse effects (AEs) will be recorded by the operator, with immediate symptomatic treatment and subsequent analysis for causation. Severe AEs will be immediately documented and reported to the Ethics Committee of the Affiliated Hospital of Chengdu University of Traditional Chinese Medicine within 24 h.

We will use an acupuncture sensation scale to assess “deqi” sensations, including soreness, numbness, heaviness, fullness, pain, and anxiety. Participants will also be able to describe any additional sensations they experience. Each sensation will be rated on a 0–10 scale, where 0 indicates no sensation and 10 represents the most intense sensation. Participants will complete this scale after each session, which will facilitate systematic collection and analysis of “deqi” sensations.

## 7 Data analysis

Data analysis will follow the intention-to-treat (ITT) strategy and will be conducted using IBM SPSS version 26.0 software (IBM Corp, New York). A *p*-value of ≤0.05 will be considered statistically significant. To address missing data, the Last Observation Carried Forward (LOCF) method will be employed for imputation. Continuous variables will be expressed as mean ± standard deviation, while categorical variables will be presented as numbers and percentages. Categorical variables will be analyzed using the Chi-Square Test. For continuous variables that follow a normal distribution, comparisons between groups will be made using an independent samples *t*-test, while paired samples *t*-tests will be used for pre- and post-treatment comparisons within the same group. For continuous data that do not follow a normal distribution, the Mann–Whitney U test will be used, with results expressed as the median and interquartile range. *p*-values obtained from the statistical tests, both for clinical outcomes and fNIRS data, will be corrected using the false discovery rate (FDR) method to account for multiple comparisons.

The raw fNIRS data will be preprocessed using NIRSPARK software, which includes format conversion, motion correction, artifact processing, and bandpass filtering. Data analysis will be performed using a dedicated module in MATLAB (R2020a).

Previous studies have demonstrated that patients with cognitive impairment due to insomnia exhibit working memory deficits correlated with cognitive performance, alongside altered connectivity patterns between the prefrontal and temporal regions (28). These findings suggest that these regions may constitute the neural basis of cognitive impairment in this population. Furthermore, acupuncture has been shown to modulate network connectivity within the prefrontal and temporal lobes in insomnia patients (29). Therefore, in this study, we will select the prefrontal and temporal regions as the regions of interest (ROIs) for activation and functional connectivity analyses. Visual representations will be generated to facilitate intuitive presentation.

In MATLAB, the NIRS-SPM will be used for analysis, converting raw data into “.mat” format. The modified Beer–Lambert law will be applied to calculate blood oxygen concentration from the raw optical intensity data. The Topo-maker module will determine the optode configuration based on standard three-dimensional brain space positional information. Initial data checks will identify channels with potential abnormalities or noticeable noise, labeling them as bad channels.

To address physiological noise and head motion artifacts, temporal derivative distribution repair (TDDR) will be employed to remove spike artifacts and baseline shifts. Subsequently, a band-pass filter will be applied to eliminate noise from heartbeats, respiration, and Mayer waves, setting the filter range to 0.01–0.1 Hz according to the experimental paradigm.

For functional connectivity analysis, the data will be segmented into resting-state and task-state periods (0-back, 2-back). Connectivity measures, including correlation coefficients (corr), coherence (coh), and phase-locking values (PLV), will be calculated across all channels for each subject. Individual connectivity matrices will be constructed, and group-level averages will be computed for each condition. Statistical analyses, such as *t*-tests or ANOVA, will compare differences between conditions. The results will be visualized using connectivity matrices and brain network plots, and the findings will be interpreted in relation to cognitive function and task-related changes.

## 8 Management of intervention and outcome changes

We expect the trial to follow this protocol. If any change needed due to new scientific evidence, recruitment issues, or data analysis, we will seek ethics approval, inform all stakeholders. These changes will be reported transparently in the final report and publications. We will approach any modifications with caution to safeguard the integrity and reliability.

## 9 Discussion

With the aging of population, attention has been increasingly focused on common health issues among the elderly. Insomnia affecting approximately 50% of older adults (39), associated with cognitive decline strongly (40–42). Insufficient sleep leads to significant differences in cognitive scores, executive functioning, and biomarkers like cerebrospinal fluid (CSF), Aβ42, and total tau (t-tau) (43, 44). Compared to younger adults, elderly are five times more likely to receive prescriptions for hypnotic drugs (45). Drugs abuse can result in severe adverse reactions, but patients may continue long-term medication due to dependency issues. In clinical practice, the efficacy of drugs approved for treating MCI remains uncertain (46). According to a report from the Boston Medication Monitoring Center in the United States, individuals aged 65 and over experience adverse drug reactions at rates 2 to 5 times higher than younger individuals (47). Striking a balance between the benefits of medication and the risks of adverse reactions is a challenge.

Non-pharmacological therapies, such as cognitive behavioral therapy, physical exercise, and hyperbaric oxygen therapy, are recommended for treating insomnia and cognitive decline, but their limited availability, time constraints, and cost hinder their adoption (47). Acupuncture has been shown to improve sleep quality and cognitive function. Rooted in TCM theory, it is believed that the heart governs the spirit and oversees mental activities, while the brain, known as the “sea of medulla,” serves as the dwelling place for the primordial spirit. Chronic insomnia combined with cognitive decline is considered to be closely intertwined with the dynamics of the spirit, stemming from a disruption in heart spirit function and deficiencies in the brain medulla. To address this issue, the trial guided by the fundamental TCM principle of “Calm the Mind and Enhance Cognitive Function.” The acupoints were selected considering TCM theories, prior research findings, and the consensus of experienced experts, include GV20, EX-HN1, GV24, HT7, GB39, and KI4.

GV20 is an essential acupoint of the Du meridian, serving as a convergence point of the Yang meridian. This acupoint has the functions of clearing the mind, calming the spirit, and promoting meridian circulation. EX-HN1 includes 4 extra-meridian acupoint situated around GV20. Its therapeutic effects are similar to GV20, used to treat related to the head disease, such as headaches, dizziness, and mental disorders. GV24, also situated at the Du Meridian, is located 0.5 cun directly above the midpoint of the anterior hairline. It is an essential point for regulating brain function, promoting mental clarity, and harmonizing the mind and spirit. HT7, as the primary acupoint of the Heart Meridian, has significance in traditional acupuncture. The efficacy including nourishing the heart, harmonizing yin and yang, tranquilizing the mind, and inducing calming and sedative responses. It is extensively used in the treatment of heart-related conditions such as anxiety, insomnia, palpitations, and emotional imbalances. It is also a valuable acupoint for stress reduction and promoting relaxation. GB39 is the “Medulla Convergence” in TCM theory, and it is believed to regulate and nourish the brain’s medulla. KI4, located on the Kidney meridian, is associated with kidney function. It is believed to enhance physical vitality and support cognitive functions positively. Stimulation of KI4 is thought to enhance blood circulation of brain and increase alertness.

Acupuncture, a widely used external therapy, offers advantages over pharmacological treatments, such as avoiding drug toxicity and side effects (48). The efficacy of acupuncture can be explained through various mechanisms, including modulating neurotransmitter release, reducing brain tissue inflammation, eliminating free radicals, inhibiting neuronal apoptosis, and suppressing tau protein expression. Recent studies have assessed the effectiveness of acupuncture (49, 50). Nevertheless, there is a debatable viewpoint suggesting that the efficacy of acupuncture may be primarily attributed to a placebo effect (51). In this study, the effectiveness of acupuncture will be assessed using placebo control to mitigate the non-specific effects of acupuncture. Concurrently, fNIRS will be utilize to investigate the functional characteristics of brain regions in patients, identify imaging characteristics associated with the disease. Compare characteristics before and after the intervention to explore the effect of acupuncture on modulating the function of these regions.

### 9.1 Advantages and limitations

This is the first trial to evaluate the efficacy of acupuncture in CID with MCI. Placebo acupuncture is used to assess the non-specific effectiveness of acupuncture, contributing to deriving more reasonable findings. To stabilize the condition of participants, regular hypnotic medications during the study period will not be restricted. The dosage and frequency of medication will be tracked by evaluators to ensure safety. The study will provide a comprehensive evaluation to assesses effects from multiple aspects, assisting clinical practitioners in accurately assessing effectiveness.

However, there remain limitations. Firstly, due to the specificity of the intervention, blinding of acupuncturists is not feasible. To mitigate potential bias, interventions will be conducted separately in individual rooms. Evaluators responsible for assessing efficacy will be independent of the acupuncturists, data summarizers, and data analysts, ensuring blinding formation. Considering this is an exploratory study aimed at observing the efficacy of acupuncture, further research will consider increasing the sample size to enhance statistical power. Additionally, stratified analyses based on severity or dose-effect analysis could provide a more accurate assessment of acupuncture’s efficacy. Thirdly, the course of acupuncture is a critical factor affecting effectiveness. The 8-week intervention period may not be sufficient for chronic disease. Further studies may extend the acupuncture duration to develop more reasonable cycles. Additionally, acupuncturists are not blinded to the acupuncture prescription. Thus, acupuncturists will be instructed to refrain from discussing treatment plans with patients to minimize potential biases. Due to limited project funding, financial compensation in cash cannot be provided. However, participants will receive personalized health guidance related to insomnia and cognitive function for 5 months. Moreover, the control group will receive free acupuncture treatment after the study concludes. All these provisions have been outlined in the ethics approval.
